# Psychiatric comorbidities and societal demographics of population with pyromania admitted to Bronx Care: a retrospective chart review

**DOI:** 10.1192/j.eurpsy.2025.1595

**Published:** 2025-08-26

**Authors:** N. Joshaghani, R. Hartnett, L. Jimenez, V. Sharma, G. Taneja, S. Gunturu

**Affiliations:** 1 Psychiatry, Bronx care Health System, NewYork; 2 St. George University, Grenada; 3 Albert Einstein College of Medicine, Bronx, United States; 4 Dayanand Medical College, Ludhiana, India; 5 Tver state Medical University, Tver Oblast, Russian Federation; 6 Psychiatry, Bronx care Health System, Bronx, United States

## Abstract

**Introduction:**

Pyromania is an impulse control disorder where repeatedly setting up intentional fires provides instant gratification after failing to control the urge. Within the psychiatric population, 6% of the individuals meet the lifetime criteria of pyromania, making it a rare diagnosis of high medico-legal significance. We aimed to estimate the prevalence of pyromania, identify psychiatric comorbidities, substance use, and related sociodemographic factors in patients with a history of pyromania within a psychiatric center in South Bronx. We hypothesize that individuals with fire-setting behavior are more likely to have antisocial tendencies, substance use disorders, and psychiatric comorbidities, leading to increased mental health service use.

**Objectives:**

The objective of this study is to estimate the prevalence of pyromania and fire-setting behavior in a psychiatric center in South Bronx, and to examine the associated psychiatric comorbidities, substance use disorders, and sociodemographic factors. By analyzing these relationships, we aim to provide insights into the characteristics and mental health service utilization patterns of individuals with pyromania or fire-setting behavior, thereby informing future treatment strategies and interventions in urban mental health settings.

**Methods:**

We searched for patients aged 12 and older, including all genders, who were evaluated or admitted through a psychiatric center within the South Bronx between December 2013 and December 2023 for a retrospective observational study. After applying inclusion/exclusion criteria, we included 11 patients diagnosed with pyromania or fire-setting behavior (ICD-10-CM code F63.1 or ICD-9-CM code 312.33), based on evaluation notes. We extracted and analyzed the data for demographics, substance use, comorbid psychiatric, and medical diagnosis.

**Results:**

We found that pyromania diagnosis was found in individual’s age range of 12 to 59 years (Mean Age-33.36 ± 14.72 years). All individuals were single, 82% were unemployed, 72.73% were US-born, and 91% were male. Psychotic disorders, primarily schizophrenia, were diagnosed in 73%, while 64% had a substance use disorder. The group comprised 45% Hispanic and 55% African American individuals.

**Conclusions:**

Our study, conducted over ten years in the Bronx, revealed a strong association between firesetting behavior and psychiatric comorbidities, particularly psychotic disorders and substance use. Contrary to our initial hypothesis, we found no significant link between firesetting and antisocial tendencies. These findings emphasize the need for targeted interventions addressing both psychiatric and sociodemographic factors, especially in urban mental health settings like the Bronx.

**Disclosure of Interest:**

None Declared

